# Prevalence of gastro-intestinal symptoms among COVID-19 patients and the association with disease clinical outcomes

**DOI:** 10.2144/fsoa-2023-0040

**Published:** 2023-04-21

**Authors:** Hafez Al-Momani, Iman Aolymat, Muna Almasri, Sameer Alhaj Mahmoud, Safaa Mashal

**Affiliations:** 1 Department of Microbiology, Pathology & Forensic medicine, Faculty of Medicine, The Hashemite University, PO box 330127, Zarqa 13133, Jordan; 2 Department of Anatomy, Physiology & Biochemistry, Faculty of Medicine, The Hashemite University, PO box 330127, Zarqa 13133, Jordan; 3 Department of basic medical science, Faculty of Medicine, Al-Balqa’ Applied University, As-Salt, Jordan

**Keywords:** clinical outcomes, COVID-19, COVID-19 transmission, gastro-intestinal system, mortality, prevalence

## Abstract

**Aim:**

This study examined the various manifestations of COVID-19 in people's gastro-intestinal system and how gastro-intestinal involvement relates to the progression and outcome of the disease.

**Methodology:**

A questionnaire survey was used to collect data from 561 COVID-19 patients between February 6 and 6 April 2022. Laboratory data and clinical outcomes were obtained from the patients' medical records.

**Results:**

39.9% of patients presented gastro-intestinal symptoms, mainly loss of appetite, nausea, vomiting and diarrhea. Gastro-intestinal symptoms were not linked to poorer outcomes such as mortality, ICU admission or length of hospital stays.

**Conclusion:**

gastro-intestinal symptoms were common among patients and may manifest with respiratory symptoms. We recommended clinicians to watch out for gastro-intestinal symptoms as related to COVID-19 infection.

## Plain language summary

COVID-19 mainly affects the respiratory system. However, it has been previously reported that the disease can impact other organ systems, particularly the gastro-intestinal system. A prospective descriptive study design which involved 561 COVID-19 patients was performed to identify the various manifestations of COVID-19 in people's gastro-intestinal system and how gastro-intestinal involvement influenced the progression and outcome of the disease. Almost 40% of patients presented with gastro-intestinal symptoms, mainly loss of appetite, nausea, vomiting and diarrhea. However, the presence of gastro-intestinal symptoms was not linked to poorer outcomes such as mortality, ICU admission, length of hospital stays and increased mechanical intubation of COVID-19 patients.

The term “corona”, meaning “crown” in Latin, is where the name “coronavirus” (CoV) originates [1]. it causes illness in humans' respiratory systems varying from mild cold to severe respiratory distress syndrome [2]. An emerging threat to world health is posed by a newly identified coronavirus (also known as severe acute respiratory syndrome (SARS)-CoV-2 or coronavirus disease 2019 (COVID-19). During the first few months of this outbreak (late 2019 onward), the COVID-19 pandemic quickly expanded from Wuhan, China to the cities of Thailand, Japan, South Korea, Singapore and Iran [3]. Global spread of the virus followed thereafter. COVID-19 was declared to be a “pandemic” by the WHO on 11 March 2020 [4]. COVID-19 is the most terrible sickness ever to have swept the planet, after the devastating 1918 flu pandemic [3]. Health and economic devastation have resulted from the reaction to the spread of COVID-19, impacting millions of people throughout the world. It is now one of the most pressing health crisis on a global scale.

It is believed that the first human cases of COVID-19 in China were acquired from a zoonotic source by contact with infected animals sold at the Huanan wholesale seafood market in Wuhan [5]. Various animal species' genomic sequences were analyzed in depth to identify the potential viral reservoir [6,7]. A recombinant virus, being one formed between the bat coronavirus and another coronavirus of unknown origin, was hypothesized to be SARS-CoV-2 [8]. Bats are the most likely wildlife reservoir of 2019-nCov, according to an analysis based on relative synonymous codon use on many different types of animals [9].

Scientists, companies, governments, and other stakeholders throughout the world have understood from the beginning that fighting COVID-19 would require the power of science and unprecedented collaboration [10]. Vaccines in the covid-19 pandemic have been a game changer in reducing rates of SARS-CoV-2 infection and hospital admission for, and mortality with, covid-19 [11]. COVID-19 vaccination exhibited high effectiveness and safety among all individual especially in those with comorbidities [12]. Significant comorbidities, such as diabetes, hypertension, obesity, and a history of ischemic heart disease were often seen in COVID-19 patients [13]. Despite vaccine effectiveness and safety, there is still some reluctance about receiving the COVID-19 vaccine [14,15]. The post-vaccine fears of bad health consequences and the acceptance of the information conveyed by social media were the two primary factors that contributed to the majority of people choosing not to have the COVID-19 vaccination [15]. Determining the characteristics and rationale for the hesitancy in taking the vaccines, and the perceived problems associated with vaccination reluctance, has persuaded individual governments in several countries throughout the globe to establish health authorities and research panels to conduct investigations [16,17].

A broad range of respiratory symptoms, from asymptomatic to mild to moderate symptoms of pneumonia, to severe patients with dyspnea and hypoxia to critically sick with respiratory failure, shock, or multiorgan failure, are caused by COVID-19, which is largely a respiratory illness [18]. Researchers previously thought it was a single-system sickness, but new evidence confirm that COVID-19 is a multi-system disease [19]. SARS-CoV-2 penetrates host cells through ACE-2. ACE-2 is a functional receptor on cell surfaces [20]. Recent studies have shown that ACE-2 is expressed in the gut more than four-times as much as in any other tissue type, including alveolar type II cells of the lung [21,22]. Given that viral nucleic acid was found in stool samples from patients, this raised the possibility that SARS-CoV-2 could also be transmitted by the feco–oral route, causing gastro-intestinal infections [23].

Although respiratory illness is the most common symptom of this infection, it has been linked to other organs, including the gastro-intestinal system. Studies showed that patients also manifest gastro-intestinal symptoms such as diarrhea, low appetite and nausea which mainly affect the gastro-intestinal system [22,24]. In their study, Wang, Hu, Hu*et al.* [25] found that 10% of individuals who tested positive for COVID-19 had diarrhea. This was echoed by another study by Pan, Mu [24] who showed that 18% of their study subjects experienced diarrhea, vomiting or abdominal pain and that a high number of COVID-19 patients (34%) reported diarrhea when admitted to hospital. According to Wan, Li, Shen*et al.* [26], gastro-intestinal and respiratory symptoms often emerged simultaneously. Researchers Pan, Mu [24] and his colleagues, on the other hand, noted that patients who presented gastro-intestinal symptoms were admitted to hospital later from the onset of symptoms compared with patients with no obvious gastro-intestinal symptoms.

The researchers made an important observation that patients who presented digestive symptoms tend to delay their hospital admission. They could be highly contagious during this time and could spread the disease more [27]. Digestive symptoms were prevalent in the community but not necessarily due to COVID-19. Researchers however advised that health professionals must take into account the possibility that COVID-19 may well be the culprit behind the gastro-intestinal symptoms [27,28]. The prognostic consequences of gastro-intestinal symptoms on outcomes have been poorly understood. Studies have shown conflicting results about the link between gastro-intestinal discomfort and poor outcomes [29,30]. This research sets out to determine how often individuals with the SARS-CoV-2 infection had digestive issues. It also advocated assessing the link between gastro-intestinal symptoms and health outcomes in an adult population in Jordan.

This study is one of few studies considering the gastro-intestinal symptoms of COVID-19 patients in Jordan. Jordan has not been exposed to natural catastrophes and tropical illnesses in the previous decades. When compared with places like the Caribbean, which has been hit by a number of hurricanes, tornadoes, and other natural disasters and has been plagued by tropical illnesses in recent decades [31], Jordan is relatively inexperienced. Several councils, institutions, and organizations were established in these, more prone, areas to combat the proliferation of tropical infectious illnesses and ensure economic stability. It was considered that learning from these disasters would encourage the affected areas to take precautions against future health and economic catastrophes [31]. However, when COVID-19 struck Jordan, a country with few preparedness measures and little catastrophe experience, it was difficult to put together an effective healthcare system to combat the spreading pandemic. Additionally, a growth in the number of intensive care beds necessitates an increase in the number of mechanical ventilators and other hospital equipment. COVID-19 has affected many other fields than medicine, including travel, manufacturing, schools, and the workforce. Chronic illnesses account for a disproportionate share of deaths and hospitalizations in Jordan. To aid in the early detection of COVID-19 cases and the prevention of future transmission, a better knowledge of its clinical symptoms is necessary.

## Methodology

### Study design & data collection

Following clearance from an institutional review board, a prospective cohort research was conducted between 6 February and 6 April 2022, during the fourth wave of SARS-CoV-2 viral propagation in Jordan. The fourth wave of the COVID-19 pandemic began in early 2022. It was said that this wave was embedded within the wave that preceded it. The omicron mutant was responsible for 55% of new infections on January 20 in Jordan [32]. By mid-February, infections escalated vigorously to reach a peak of ∼30 000 cases per day with Omicron variant dominance.

The participants in this research are people aged 18 years or older who were hospitalized at Prince Hamza Hospital for the purposes of treatment and isolation. PCR swab testing was used to make the diagnosis of COVID-19 in those patients. Patients who had had gastro-intestinal surgery in the past, suffered from mental illness, or were less than 18 years old were not included. We interviewed all patients who were eligible for the study and who had signed a consent form, monitored their progress in the hospital, and analyzed the data from all participants.

The patients' demographic characteristics, their presenting symptoms and signs, including their gastro-intestinal symptoms, as well as their clinical and laboratory investigation results were collected. Upon hospital admission, patients were interviewed using a pre-designed, two-part questionnaire. The interview was conducted with the assistance of a trained nurse who collected information such as patient demographics, comorbid conditions, gastro-intestinal symptoms, respiratory symptoms, gustatory symptoms, and olfactory symptoms.

The first part of the questionnaire consisted of patient demographics and their medical and surgical histories, as well as their medication histories. The researchers calculated the patients' BMI based on self-reported height and weight data.

The second part of the questionnaire consisted of a symptom evaluation, and patients were asked about their major complaints. They were also asked whether they experienced any of the listed symptoms. The listed symptoms were divided into three categories, namely: general symptoms; respiratory symptoms; and gastro-intestinal symptoms. The researchers gathered laboratory data and patient clinical outcomes, such as intensive care unit (ICU) admission, the need for intubation and mechanical ventilation, and the mortality rate, from the patients' medical records.

### Patient classification

According to the Guidance for COVID-19 that was published by the National Institutes of Health [33], each of the patients who participated in the research was assigned to one of three different groups based on the severity of their illness, which were as follows:

- Mild illness: a patient who does not have shortness of breath, or abnormal chest x-ray or CT scan findings, but who exhibits symptoms consistent with COVID-19.
- Moderate illness: Patients whose oxygen saturation (SpO_2_) less than 94% and who have abnormal radiological results from a chest x-ray or CT scan indicating lower respiratory illness.
- Severe illness: In addition to abnormal radiological findings from chest x-rays or CT scans, patients with severe symptoms such as a respiratory rate exceeding 30-times per minute, pulse oxygen saturation levels of 93% or below or rapid pneumonia progression within 24 to 48 hours.

### Outcomes

The incidence of gastro-intestinal symptoms at initial presentation in patients with COVID-19 was considered the primary outcome. Secondary analyses were made with regards to associations between gastro-intestinal symptoms and general and respiratory symptoms, laboratory results, patient characteristics, and the length of hospitalization.

### Statistical Analysis

To conduct the analysis of the study data, the researcher used GraphPad InStat 6.0. For continuous data, means ± standard deviation was calculated based on patient characteristics, COVID-19 manifestations, laboratory data, and hospitalization outcomes. For categorical data, frequencies and proportions were calculated based on patient characteristics and COVID-19 manifestations. Both the Chi-square test and Fisher's exact test were utilized to compare the categorical data, while the two-sample *t*-test and the Wilcoxon rank-sum test were used to compare the continuous data [12]. Logistic regression was used in the process undertaking multivariable analyses. To determine significant predictors of the gastro-intestinal manifestations of COVID-19 and hospitalization outcomes, logistic regression analyses were carried out. The results of logistic regression analyses were reported as odds ratios (OR) with corresponding 95% confidence intervals (CIs), considering factors such as age, sex, BMI, comorbidities, and the presence of respiratory and constitutional symptoms. These analyses were conducted to determine significant predictors of the gastro-intestinal manifestations of COVID-19 and hospitalization outcomes. In this study, statistical significance was determined to exist when the p value was less than 0.05.

## Results

### Patient's demographic characteristic

The study involved a total of 561 patients who tested positive for COVID-19. The patients were mostly overweight to obese, with a mean BMI of 29.0 ± 4.5 kg/m^2^. Many of them presented certain cardiovascular risk factors, including coronary artery disease, congestive heart failure, cardiac arrhythmia, and other comorbidities. The most common gastro-intestinal comorbidities among the patients were gastroesophageal reflux disease (18.7% [n = 105]) followed by irritable bowel syndrome (8.4% [n = 47]), inflammatory bowel disease (3.0% [n = 17]) and peptic ulcer disease (6.4% [n = 36]). There was no difference when it came to baseline demographics between those with gastro-intestinal symptoms and those without (Table 1).

**Table 1.** Clinical and demographic characteristics of COVID-19 patient cohort.

Patient characteristics	All patients (n = 561)	Patient with no gastro-intestinal symptoms (n = 337, 60.1%)	Patient with gastro-intestinal symptoms (n = 224, 39.9%)	p-value
Age, years, mean ± SD	61.4 ± 15.6	60.3 ± 11.9	62.0 ± 13.6	0.46
Female, n (%)	302 (53.8%)	173 (51.3%)	129 (57.5%)	0.38
BMI, kg/m^2^, mean ± SD	29.0 ± 4.5	30.5 ± 6.7	29.3 ± 6.2	0.21
Past medical history, n (%)				
Coronary artery disease	123 (21.9%)	71 (21.1%)	52 (23.2%)	0.37
Congestive heart failure	74 (13.2%)	47 (13.9%)	27 (12.1%)	0.45
Cardiac arrhythmia	87 (15.5%)	54 (16.0%)	33 (14.7%)	0.37
Hypertension	149 (26.5%)	88 (26.1%)	61 (27.2%)	0.42
Hyperlipidemia	123 (21.9%)	72 (21.4%)	51 (22.8%)	0.80
Diabetes	109 (19.4%)	66 (19.6%)	43 (19.2%)	0.78
Cerebrovascular accident	122 (21.7%)	74 (22.0%)	48(21.4%)	0.64
Pulmonary disorders	99 (17.6%)	58 (17.2%)	41 (18.3%)	0.57
Chronic renal insufficiency	62 (11.1%)	36 (10.7%)	26 (11.6%)	0.32
Thyroid disorders	41 (7.3%)	21 (6.2%)	20 (8.9%)	0.66
Gastroesophageal reflux disease	105 (18.7%)	67 (19.9%)	38 (17.0%)	0.91
Irritable bowel syndrome	47 (8.4%)	31 (9.2%)	16 (7.1%)	0.34
Inflammatory bowel disease	17(3.0%)	11 (3.3%)	6 (2.7%)	0.52
Peptic ulcer disease	36 (6.4%)	22 (6.5%)	14 (6.3%)	0.61
Other GI disorders	25 (4.5%)	16 (4.7%)	9 (4.0%)	0.24
Social history				
Alcohol use	21 (3.7%)	11 (3.3%)	10 (4.5%)	0.25
Tobacco use	113 (20.1%)	64 (19.0%)	49 (21.9%)	0.67

### Prevalence of gastro-intestinal symptoms

**Figure 1.**
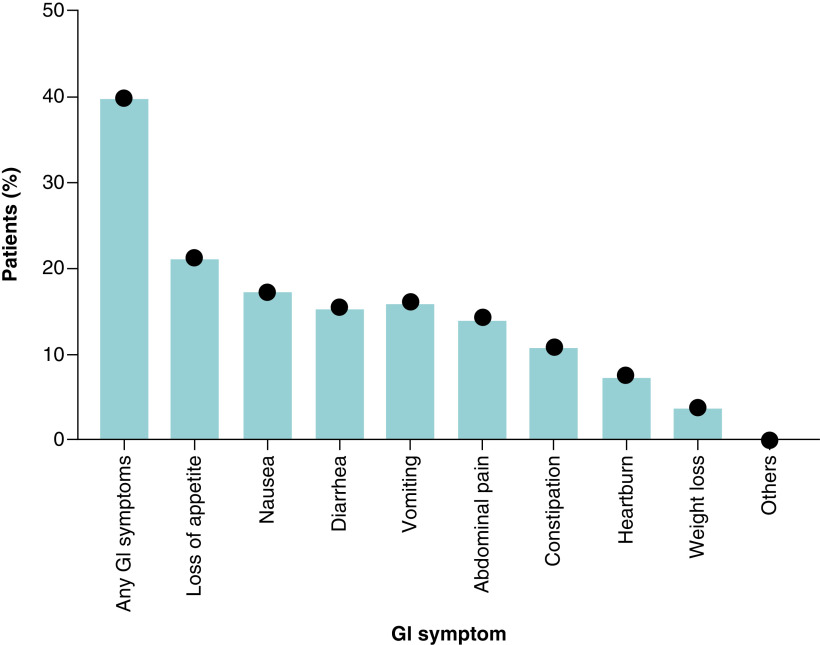
Gastro-intestinal symptoms reported by the COVID-19 patients included in this study, x-axis represent the symptoms and y-axis represent the percentage of patient reported each symptom of the total 561 COVID-19 cohort.

The study showed that about 39.9% of patients reported at least one gastro-intestinal symptom on presentation. The most common gastro-intestinal symptom reported was loss of appetite (21.2%) followed by nausea (17.3%) and then vomiting and diarrhea (16.2% and 15.5%, respectively). Other gastro-intestinal symptoms presented were abdominal pain (14.3%), constipation (10.9%) and heartburn (7.6%) (Figure 1). Gastro-intestinal symptoms were not the patients' principal presenting complaint of COVID-19 among all the subjects of the study.

### Gastro-intestinal symptoms & patients' clinical presentation

Findings of this study showed that patients with gastro-intestinal symptoms reported statistically significantly lower rates of fatigue (59.4% vs 76.3%; p < 0.0001) and myalgia (27.7% vs 57.3%; p < 0.0001) (Table 2). Moreover, patients with gastro-intestinal symptoms reported statistically significantly lower rates of some respiratory symptoms, including cough (80.4% vs 86.9%; p = 0.04) and shortness of breath (58.0% vs 72.4%; p < 0.0001). However, patients with gastro-intestinal symptoms reported significantly higher rates of loss of taste (53.3% vs 35.9%; p < .0001). No other gastro-intestinal or respiratory symptoms were found to be statistically different between those with and without gastro-intestinal symptoms. The study also observed that arthralgia, sore throat, and loss of smell were more common among patients with gastro-intestinal symptoms, but not at a statistically significant level.

**Table 2.** General and respiratory symptoms reported by the COVID-19 patients included in this study.

Symptoms	All patients (n = 561)	Patient with no gastro-intestinal symptoms (n = 337, 60.1%)	Patient with gastro-intestinal symptoms (n = 224, 39.9%)	p-value
General symptoms				
Fever	492 (87.7%)	302 (89.6%)	190 (84.8%)	0.11
Fatigue	390 (69.5%)	257 (76.3%)	133 (59.4%)	<0.0001^†^
Myalgia	255 (45.5%)	193 (57.3%)	62 (27.7%)	<0.0001^†^
Chills	156 (27.8%)	103 (30.6%)	53 (23.7%)	0.08
Arthralgia	45 (8.5%)	22 (6.5%)	23 (10.3%)	0.11
Respiratory symptoms				
Cough	473 (84.3%)	293 (86.9%)	180 (80.4%)	0.04^†^
Shortness of breath	374 (66.7%)	244 (72.4%)	130 (58.0%)	<0.0001^†^
Sore throat	295 (52.6%)	174 (51.6%)	121 (54.0%)	0.60
Sputum production	179 (31.9%)	106 (31.5%)	73 (32.6%)	0.78
Rhinorrhea	93 (16.6%)	61 (18.1%)	32 (14.3%)	0.25
Loss of smell	272 (48.5%)	158 (46.9%)	114 (50.9%)	0.38
Loss of taste	206 (36.7%)	121 (35.9%)	119 (53.3%)	<0.0001^†^

† Statistically significant with p < 0.05.

### Gastro-intestinal symptoms & patients' laboratory test results

This study found no significant differences in any of the routine tests done at the time of hospital admission (Table 3). Among the tests performed were blood tests (e.g., leukocyte count, hemoglobin level, and platelet count), coagulation tests (international normalized ratio (INR), partial thromboplastin time (PTT)), liver enzymes (including: aspartate aminotransferase (AST), alanine aminotransferase (ALT), alkaline phosphatase, and total bilirubin), and the cardiac enzyme creatinine kinase between patients with and without gastro-intestinal symptoms. Inflammatory markers that were tested in this study were ferritin, D-dimer, and C-reactive protein.

**Table 3.** Baseline laboratory values on admission of COVID-19 patient cohort.

Laboratory test	All patients (n = 561)	Patient with no gastro-intestinal symptoms (n = 337,60.1%)	Patient with gastro-intestinal symptoms (n = 224,39.9%)	p-value
Blood tests				
White blood cell count, 10^9^/l	10.8 ± 11.2	9.3 ± 9.7	9.5 ± 10.2	0.44
Hemoglobin, g/dl	13.3 ± 32.9	13.6 ± 4.1	12.8 ± 4.3	0.75
Platelets, 10^9^/l	208.5 ± 90.7	218.6 ± 80.1	213.2 ± 87.8	0.42
Coagulation tests				
INR	1.2 ± 0.59	1.3 ± 0.64	1.1 ± 0.6	0.67
PTT, seconds	37.8 ± 15.9	35.8 ± 11.6	37.8 ± 18.8	0.43
Liver enzymes				
Aspartate aminotransferase (AST), u/l	40.2 ± 34.2	45.6 ± 45.3	45.7 ± 34.2	0.16
Alanine aminotransferase (ALT), u/l	49.0 ± 30.1	42.9 ± 21.1	42.1 ± 25.7	0.77
Alkaline phosphatase, U/L	75.5 ± 38.3	80.1 ± 48.1	77.2 ± 48.8	0.87
Total bilirubin, mg/dl	0.62 ± 0.39	0.45 ± 0.50	0.66 ± 0.22	0.23
Amylase (serum) units/l	98 ±66.5	109 ± 44.2	120 ± 24.8	0.16
Cardiac markers				
Creatine kinase u/l	350 ± 189	388 ± 120	312 ± 167	0.58
Inflammatory markers				
D-dimer, ng/ml	240 ± 125	319 ± 190	280 ± 180	0.43
C-reactive protein, (mg/dl)	8.7 ± 3.2	8.8 ± 4.2	0.9 ± 4.5	0.54

### Gastro-intestinal symptoms & patients' Clinical outcome

Among the study population, 11.7% of patients (n = 66) were admitted to the ICU, 8% (n = 45) were intubated, 3.3% (n = 19) died in the hospital, and the mean length of stay for all patients was 8.2 ± 3.9. As shown in Table 4, the influence of gastro-intestinal symptoms on clinically important outcomes was assessed by a multivariable logistic regression analysis. After adjusting for any confounding factors, gastro-intestinal symptoms did not seem to have a statistically significant role in determining patients' clinical results. However, there is a reduced risk for ICU admission (OR: 0.81 [95% CI: 0.41 to 1.75]; p = 0.87), mechanical ventilation (OR: 0.92; 95% CI: 0.50 to 2.04]; p = 0.52) among patients with gastro-intestinal symptoms.

**Table 4.** Clinical and hospitalization outcomes of COVID-19 patient cohort.

Outcome	All patients (n = 561)	Patient with no gastrointestinal symptoms (n = 337 [60.1%])	Patient with gastrointestinal symptoms (n = 224 [39.9%])	Adjusted odds ratio (ORa)	p-value
Length of hospital stay, mean (SD)	8.2 ± 3.9	7.9 ± 4.9	8.3 ± 2.7		0.54
ICU stay, n (%)	66 (11.7)	39 (11.5)	27 (12.0)	0.81 (0.41–1.75)	0.87
Mechanical ventilation, n (%)	45 (8.0)	26 (7.7)	19 (8.4)	0.92 (0.50–2.04)	0.52
Death, n (%)	19 (3.3%)	9 (2.6)	10 (4.4)	1.07 (0.57–3.8)	0.27

### The correlation of the presence of gastro-intestinal symptoms with the severity of COVID-19 infection

The presence of gastro-intestinal symptoms was a predictor of a less severe COVID-19 infection (Table 5). Gastro-intestinal symptoms tend to be more related to the milder form of COVID-19 infection, with an OR of having a mild illness in a patient with gastro-intestinal manifestations (OR: 1.72 [95% CI: 1.19 to 2.20]; p = 0.01). Gastro-intestinal manifestations among COVID-19 patients appeared to be protective factors for the development of a moderate level of COVID-19 infection (OR: 0.55 [95% CI: 0.46 to 1.01]; p = 0.03). While gastro-intestinal symptoms are neither predictors nor protective of the development of the severe form of COVID-19 infection (OR: 1.05 [95% CI: 0.62 to 1.75]; p = 0.88).

**Table 5.** The association between gastrointestinal manifestation and Ccovid-19 infection severity.

	Patient with no gastrointestinal symptoms (n = 337 [60.1%])	Patient with gastrointestinal symptoms (n = 224 [39.9%])	Adjusted odds ratio (ORa)	p-value
Mildly ill group (n = 187,33.3%)	93 (27.6%)	94 (42.0%)	1.72(1.19–2.2)	0.01
Moderately ill group (n = 308, 54.9%)	205 (60.8%)	103 (46.0%)	0.55 (0.46–1.01	0.03
Severely ill group (n = 66,11.8%)	39 (11.6%)	27 (12.0%)	1.05 (0.62–1.75)	0.88

## Discussion

This study showed that of the 561 patients who were hospitalized with COVID-19, 39.9% manifested gastro-intestinal symptoms including diarrhea, nausea, vomiting, and abdominal pain. “Loss of appetite” came at the top of the list, followed by nausea or vomiting and diarrhea, 7.6% of the patients had heartburn. The researchers found no significant differences between patients with and without gastro-intestinal symptoms with regards to patient demographic characteristics or their comorbid diseases. In this cohort of patients, gastro-intestinal symptoms were not the initial presentation symptoms.

Since the outbreak of COVID-19 in Wuhan, China, more and more people, particularly health workers, are becoming aware of gastro-intestinal manifestations in COVID-19 patients. When COVID-19 was first detected in Wuhan, diarrhea was reported in only 3% of the cases [28]. However, subsequent studies from Wuhan showed diarrhea cases to rise to 10% and to 25% of all cases in a study from Singapore [25,34]. As health workers became more aware of gastro-intestinal manifestations in COVID-19 patients, reports of gastro-intestinal symptoms in subsequent studies also increased. One study found a higher frequency (39.6%) of gastro-intestinal symptoms, with nausea being reported as the most prevalent symptom [35]. Meanwhile, Pan, *et al.*’s Chinese-based research [24] indicates that more than half of the study's patient population experienced gastro-intestinal symptoms.

Our own study showed a prevalence of gastro-intestinal symptoms at 39.9% among COVID-19 patients, which was considerably lower than that reported by other studies from the USA and China, which ranged from 50.5% to 61.3% [24,36,37]. In such studies, the inclusion of anorexia in the list of gastro-intestinal symptoms may explain the higher reported incidence of gastro-intestinal symptoms overall. However, the present study did not include anorexia as a gastro-intestinal symptom since it is non-specific and may alternatively be associated with other infectious or inflammatory processes. A study in Wuhan, China, reported that 50% of their subjects had gastro-intestinal symptoms, but most of these patients (78.6%) had anorexia [24]. However, when anorexia was taken out of the equation, only 18.6% came up as having specific gastro-intestinal symptoms.

The most common gastro-intestinal symptoms found in our study were loss of appetite (21.2%) and nausea (17.3%) followed by vomiting, diarrhea, and abdominal pain (16.2, 15.5 and 14.3%, respectively). A study in Wuhan, China, by Damaso, Oliveira, Massarani, Moussatché [38] showed similar results, wherein almost half, or 49.5% of the 305 COVID-19 patients reported having had diarrhea. The same study reported an incidence of nausea at 29.4%, vomiting at 15.9% and abdominal pain at a relatively low 6.0%. Other studies from Wuhan that involved COVID-19 patients reflected similar outcomes, showing nausea, vomiting, and diarrhea as the main gastro-intestinal symptoms among patients [25,39,40].

Our research has shown that heartburn is experienced by 7.6% of patients who have gastro-intestinal symptoms, and that 18.7% of our patient cohorts had the comorbidity of gastroesophageal reflux (GOR) disease. Since ACE-2 through which SARS-CoV-2 enters the host cells is highly expressed in upper part of gastro-intestinal tract [41], we might postulate that (micro)aspiration through GOR is a possible pathway for the transmission of SARS-CoV-2 from the stomach to the lungs. GOR is a common condition defined by a retrograde flow of stomach contents into the esophagus [42,43]. GOR may be a physiological process that many individuals experience routinely, but it can also be pathological, leading to GOR disease characterized by an abnormally high level of GOR [44,45].

Evidence of an infectious virus, not only viral RNA, was found in the stool of a patient with severe COVID-19 illness, according to a study from the US Centers for Disease Control and Prevention (CDC) [46]. The discovery of ‘live’ viruses in human stool was also reported in a separate investigation [47]. According to other research, approximately 50% of COVID-19 patients have detectable viral RNA in their feces, even when the virus is not present in other organ systems [48]. This may indicate the gastro-intestinal system as a major portal for SARS-CoV-2 infection with the possibility of fecal–oral or gut to lung transmission. Further research is warranted on the possibility that SARS CoV-2 is being aspirated into the lungs from a stomach reservoir, as suggested by our data.

Our prior studies have hinted at a potentially substantial stomach infection etiology in cystic fibrosis (CF), as well as a potential reservoir of microorganisms [49,50]. This link between gastric juice microflora and sputum samples from cystic fibrosis (CF) patients has revealed a correlation between the two [49,50]. Furthermore, supporting the concept of microbial transmission between the respiratory and gastro-intestinal systems is evidence from a study of CF patients in children by Palm, Sawicki, and Rosen [49] showing a link between lower-airway *pseudomonas aeruginosa* infection and GOR. Independent of the flora in the oropharynx, another study also revealed microbial translocation from the gastro-intestinal tract to the lungs [51]. The importance of GOR and the potential function of microaspiration in the spread of COVID-19 has never previously been investigated. Therefore, further studies are needed.

Our own study showed that gastro-intestinal symptoms were not linked to poorer outcomes such as increased mortality, a longer hospital stay, or increased mechanical intubation of COVID-19 patients, with the tendency toward a milder form of infection in patients with gastro-intestinal symptoms. We also found no difference in the results of laboratory tests between patients with gastro-intestinal symptoms and those without. Earlier studies have reported conflicting findings with regards to the presence of gastro-intestinal symptoms and poor outcomes. One study from Wuhan, China showed that patients who had digestive symptoms had longer hospital stays (9 vs 7.3 days, p = 0.013). Another study by Zhou *et al.* [52] showed that as the severity and duration of COVID-19 increased, gastro-intestinal symptoms increased as well. In a multicenter study of 191 patients [52], the presence of gastro-intestinal symptoms was associated with elevated C-reactive protein, elevated alanine aminotransferase, and lower hemoglobin levels when compared with patients without gastro-intestinal symptoms. However, another study by Redd [36], showed no difference in clinical outcomes in patients with or without gastro-intestinal symptoms. It also showed no significant differences in the leukocyte count, hemoglobin, platelets, coagulation, and liver tests among patients with or without gastro-intestinal symptoms [36]. This disparity between different studies could be related to different study design and sampling approaches as well as the variation in clinical features of different SARS-CoV-2 variants.

There are still gaps in our knowledge as to what specific processes caused gastro-intestinal manifestations in COVID-19. There are many proffered theories. One theory touched on the fact that intestinal tropism has been associated with SARS-CoV-2, which could be due to its strong affinity for ACE-2 receptors. ACE-2 receptors are highly expressed in the esophagus and intestinal epithelial cells [53]. Hence, there is a strong possibility of direct small bowel involvement resulting in direct cytopathic effects causing gastro-intestinal symptoms. Similar to another study [36], we found that loss of smell (anosmia) and loss of taste (ageusia) were commonly found among patients with gastro-intestinal symptoms. While the exact cause of this association is unclear, it could be due to damage to olfactory and gustatory receptors during viral entry through nasal and oral routes [53]. In a separate study from Hong Kong, patients with diarrhea on presentation had higher rates of stool RNA positivity when compared with those without diarrhea (38.5 vs 8.7%, p = 0.02). This implies a direct effect of SARS-CoV-2 on the gastro-intestinal tract [54]. The viral infection can cause altered intestinal permeability, resulting in malabsorption [55]. Another theory suggested that the inflammatory response from a cytokine storm in severe COVID-19 patients can cause hypoxia-induced bowel ischemia, resulting in diarrhea and other gastro-intestinal symptoms.

## Limitations

There are some limitations to this study, one of which is the lack of a validated symptom questionnaire survey. Another was that it was limited to a single-center hospital-based study, which could result in regional bias. We advise other researchers to exercise caution before generalizing our experience. Moreover, the presence of SARS-CoV-2 RNA in stool could not be correlated with gastro-intestinal symptoms because the relevant test was not routinely performed at the researchers' institution.

In addition, this study did not use a control group composed of patients who tested negative for COVID-19, which the researchers acknowledge would have provided further empirical insight into the representation of gastro-intestinal symptoms in patients with COVID-19. However, notwithstanding these limitations, this study has certain strengths, including its prospective design using a predesigned questionnaire and the large study sample. The researchers believe that these factors overcome some of the inherent bias found in studies that focus on retrospective chart reviews.

The significance and implications of this study lie in its confirmation of the involvement of the gastro-intestinal system in COVID-19 infection, which can mimic a range of gastro-intestinal differential diagnoses. In patients with gastro-intestinal symptoms, failing to acknowledge the possibility that the patient is infected with COVID-19 may not only lead to diagnostic issues, but also failing to isolate the patient accordingly can prolong the spread of the disease [56]. Such isolation delays risk unnecessarily exposing people to the COVID-19 virus, including the patient's family members and friends, other hospital patients, and clinical staff. Failing to identify COVID-19 infection in gastro-intestinal symptomatic patients can also impact virus transmission as a result of underestimating the incidence of the virus. Moreover, finding of this study suggested that there is a need for further studies to explore the role of GOR and aspiration in the transmission of COVID-19 and the impact of reflux on lung involvement.

## Conclusion

We found that gastro-intestinal symptoms were routinely encountered among hospitalized COVID-19 patients. Certain gastro-intestinal symptoms such as loss of appetite, nausea, vomiting, and diarrhea were found to be widely prevalent among COVID-19 patients. This study showed that the presence of gastro-intestinal symptoms was not linked to poorer outcomes such as increased mortality, longer hospital stays, or increased mechanical intubation of COVID-19 patients. It seemed that gastro-intestinal symptoms could potentially be a precursor or determining factor in the diagnosis of COVID-19. The authors of this study recommend that more extensive studies be made to evaluate the effects of gastro-intestinal symptoms on COVID-19 outcomes and that epidemiologic studies involving a larger population sample should be carried out to further confirm this study's findings.

**Summary points**

- COVID-19 is a new disease that primarily affects the respiratory system However, it was found to impact on other organ systems particularly the gastro-intestinal system.
- The study showed that 39.9% (244/561) of patients involved in the study presented gastro-intestinal symptoms.
- The most common gastro-intestinal symptoms were loss of appetite, nausea, vomiting and diarrhea.
- The presence of gastro-intestinal symptoms was not linked to poorer clinical we recommended for clinicians to watch out for gastro-intestinal symptoms and to be aware of the fecal–oral route of transmission so that precautionary measures may be established to prevent further development of the disease.
